# Complete and durable response to crizotinib in a patient with malignant pleural mesothelioma harboring *CD74*-*ROS1* fusion

**DOI:** 10.1007/s00432-022-04076-0

**Published:** 2022-06-01

**Authors:** Xuehua Xie, Mengxing You, Erhong Meng, Shunyou Wang, Beifang Niu, Weiming Huang

**Affiliations:** 1 Department of Medical Oncology, The First Hospital of Putian, Teaching Hospital, Fujian Medical University, Putian, China; 2 Computer Network Information Center, Chinese Academy of Sciences, Beijing, China; 3 ChosenMed Technology (Beijing) Co., Ltd., Beijing, China; 4 University of the Chinese Academy of Sciences, Beijing, China

**Keywords:** Malignant pleural mesothelioma, CD74-ROS1 fusion, Crizotinib, Targeted therapy

## Abstract

Malignant pleural mesothelioma (MPM) is a rare and deadly malignancy with an extremely poor prognosis. The median overall survival (OS) of this disease is 12–18 months. However, the oncogenic driver mutations of MPM are rarely understood, and the targeted therapy for it is still under investigation. In this report, we describe a case of MPM with *CD74-ROS1* fusion who obtains complete and durable response after receiving crizotinib. By the time of submission, the progression-free survival (PFS) with crizotinib has been 6.0 years, and the patient has survived for 7.6 years. Currently, he is still in complete remission (CR). To the best of our knowledge, this case represents the first report of *CD74-ROS1* fusion identified in MPM. Meanwhile, it is also the first report of complete and long-term response to crizotinib in a patient with MPM positive for *CD74-ROS1* fusion. This case report might contribute to the tumorigenesis and targeted therapy of this deadly disease.

Malignant pleural mesothelioma (MPM) is a rare and highly deadly cancer. Its prognosis is extremely poor, with a reported median overall survival (OS) of 12–18 months, and no definitive therapy is available for this lethal disease. Moreover, MPM is refractory to the trimodal therapy consisting of chemotherapy, radiotherapy and surgery (Scherpereel et al. 2018). To date, there has not been approved targeted therapy for it. Over the recent years, advances in the fields of genomics and functional genomics have achieved a breakthrough in the complex genetic landscape of MPM. However, limited information is available regarding gene fusions in this fatal disease. ROS proto-oncogene 1 (*ROS1*) fusion is one of oncogenic driver mutations in 1–2% of patients with non-small-cell lung cancer (NSCLC), with *CD74-ROS1* fusion being the most common one in light or non-smokers (Bergethon et al. 2012). In 2016, the Food and Drug Administration (FDA) of the United States approved crizotinib as first-line therapy for *ROS1*-positive advanced NSCLC. However, to date, *CD74-ROS1* fusion and the corresponding targeted therapy have not been reported in patients with MPM. Herein, we describe the first case of MPM with *CD74-ROS1* fusion who achieves complete and long-term response after receiving treatment with crizotinib.

In 2014, a 19-year-old-male patient was admitted to a local hospital due to chest pain without obvious inducement. Thoracic computed tomography (CT) showed a space-occupying lesion under the left upper pleura (Fig. 1A). Preliminary diagnosis was left posterior mediastinal tumor, more likely to be malignant.

**Fig. 1:**
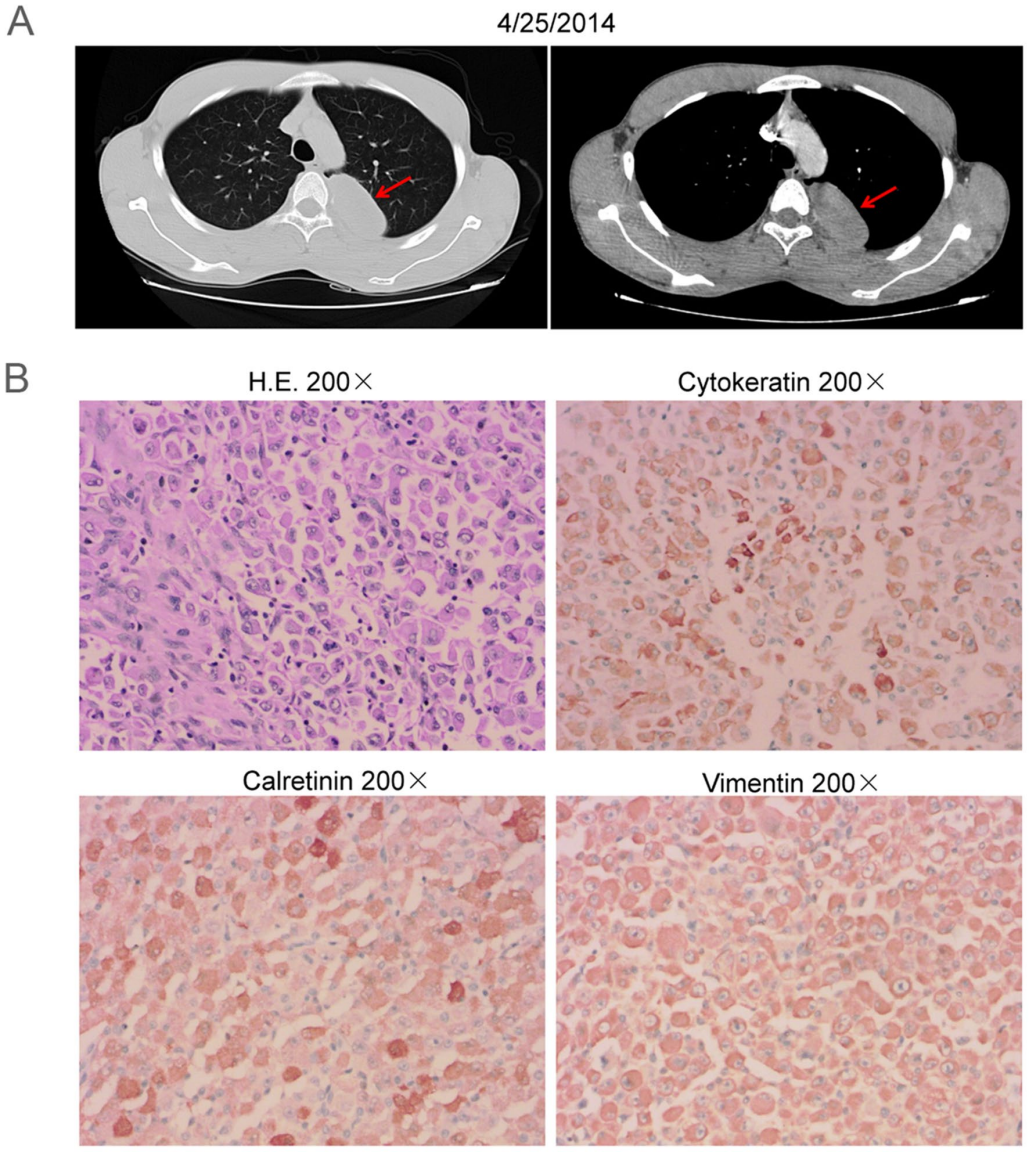
Radiographic imaging at diagnosis and pathology. **A** Computed tomography (CT) showed a space-occupying lesion under the left upper pleura. **B** Hematoxylin and eosin (H.E.) staining revealed the space-occupying lesion was malignant pleural mesothelioma (MPM). Immunohistochemistry (IHC) showed that tumor cells were positive for cytokeratin, calretinin and vimentin

On April 29, 2014, the patient underwent video-assisted thoracoscopic surgery (VATS) and thoracoscopic wedge resection (TWR) of the left upper lobe. Postoperative pathology showed the space-occupying lesion was epithelioid malignant pleural mesothelioma invading pulmonary parenchyma. Meanwhile, tumor thrombus in vessels was observed. Immunohistochemistry (IHC) showed that tumor cells were positive for cytokeratin, vimentin, calretinin, and negative for alpha smooth muscle actin (α-SMA), desmin, MyoD1, myogenin, p63, CD45, CD38, CD138, S-100, C5/6, mesothelial cells (Fig. 1B). The final diagnosis was stage II MPM (pT2NxM0). Subsequently, he received six cycles of postoperative adjuvant chemotherapy combined with pemetrexed and cisplatin, and no adverse events were reported from him.

In July 2015, hoarseness was developed in this patient. Meanwhile, thoracic CT showed enlargement of mediastinal lymph node indicating relapse. On September 10, 2015, he received six cycles of first-line chemotherapy combined with pemetrexed and carboplatin. After two cycles, thoracic CT showed the mediastinal lymph node reduced from 3.94 cm × 4.16 cm in size to 1.0 cm in diameter. Therefore, the clinical response was evaluated as partial remission (PR). However, after six cycles, thoracic CT showed that the mediastinal lymph node was enlarged indicating progressive disease (PD).

To seek personalized therapy strategies, paraffin-embedded sections of tumor tissues resected from the patient were subjected to real-time PCR (RT-PCR), and the results showed there were no EGFR mutations, MET amplifications or ALK fusions, but *ROS1* fusion identified in the patient. However, the partner type of *ROS1* could not be determined through this method. From February 2016, he began to receive second-line therapy with oral crizotinib. On June 22, 2016, thoracic CT revealed that the mediastinal lymph node shrank remarkably indicating PR. Furthermore, on December 27, 2020, chest CT scan showed that the mediastinal lymph node disappeared suggesting complete remission (CR). By the time of submitting this manuscript, the progression-free survival (PFS) of second-line therapy with oral crizotinib has been 6.0 years, and he has survived for 7.6 years. Therefore, the patient achieved complete and durable remission (Fig. 2). At present, the patient is still in CR.

**Fig. 2:**
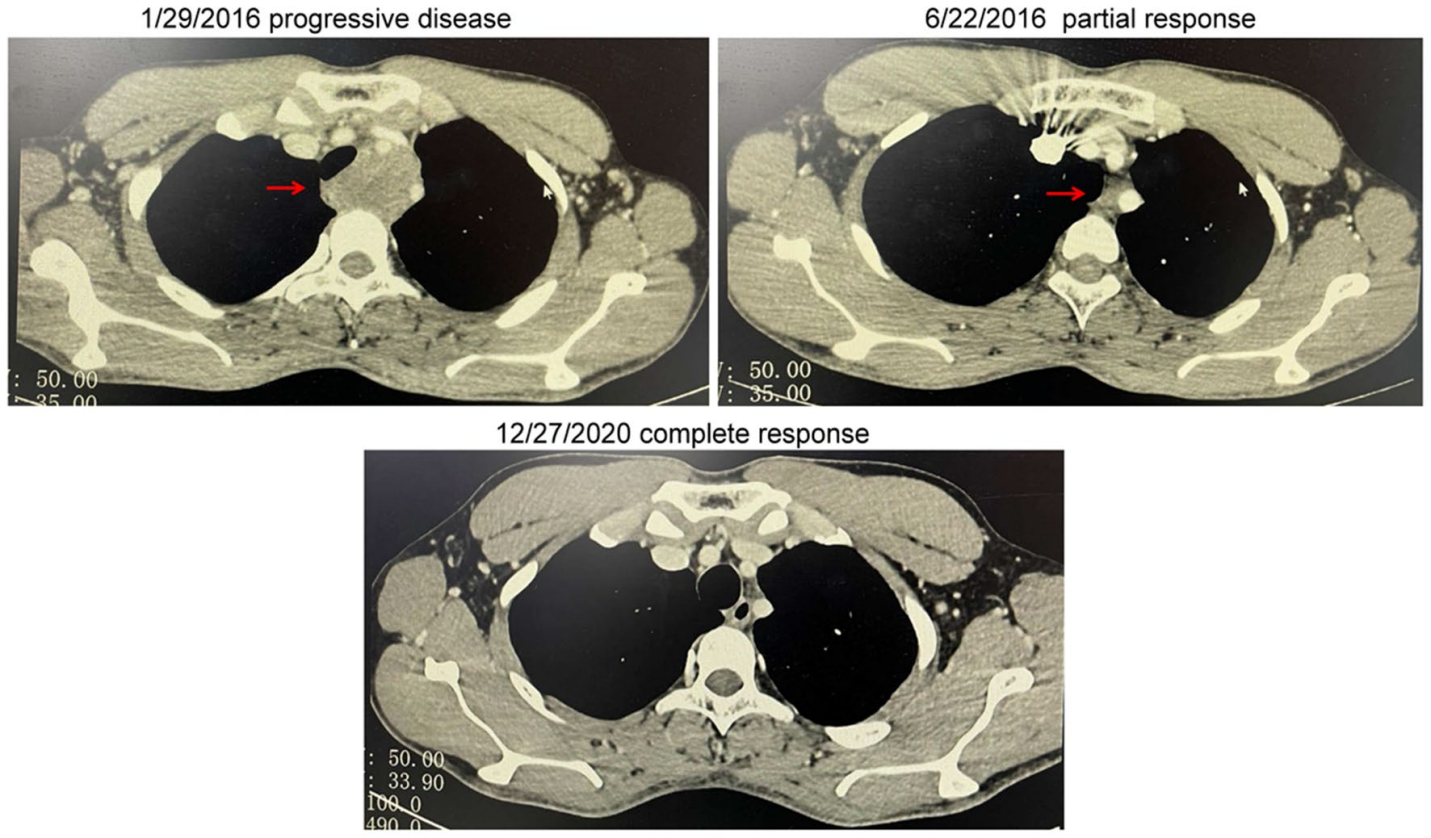
Dynamic imaging of mediastinal lymph node at different stages of the treatment. The mediastinal lymph node markedly shrank and finally disappeared after treatment with crizotinib

To find out the partner type of *ROS1* in the patient, paraffin-embedded sections of tumor tissue resected from him were subjected to next-generation sequencing (NGS) through a 599-gene panel (ChosenMed Technology [Beijing] Co. Ltd, Beijing, China) on August 12, 2021. The results revealed he harbored *CD74-ROS1* fusion (Fig. 3A). This fusion included exons 1–7 of *CD74* and exons 33–43 of *ROS1*, which retained the complete tyrosine kinase domain of *ROS1* (Fig. 3B). The somatic and germline mutations in the patient were showed in Tables 1 and 2, respectively. The timeline of the patient was demonstrated as Fig. 3C.

**Fig. 3:**
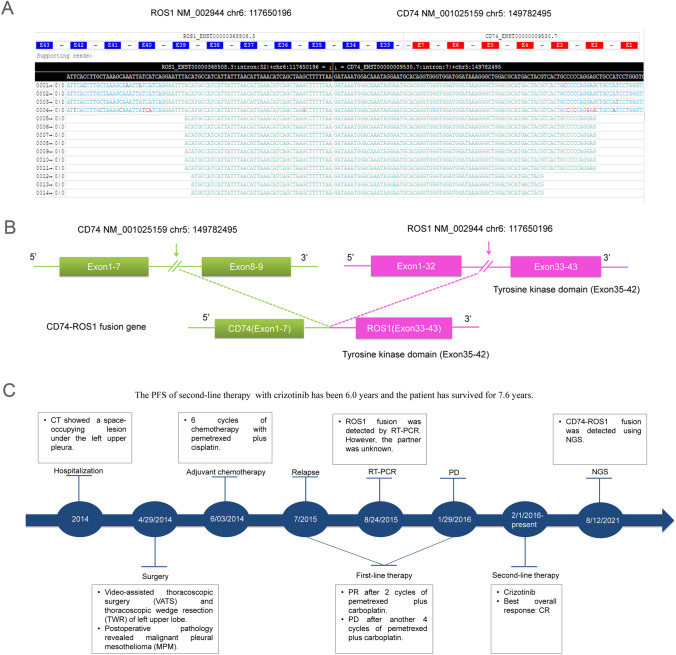
Next-generation sequencing findings of the primary malignant pleural mesothelioma tissue samples and case timeline. **A** Visualization of the *CD74-ROS1* fusion using the Integrative Genomics Viewer browser (IGV). **B** An intergenic region between *CD74* exon 1–7 and ROS proto-oncogene 1 (*ROS1*) exon 33–43 fusion variant was identified. **C** The timeline of the patient

**Table 1:** Somatic mutations in the patient

Gene	Transcript	Exon	Nucleotide change	Alteration	Mutant allele frequency	Variation type
CD74-ROS1 fusion	NM_001025159	1–7	CD74 (exon 1–7)—ROS1(exon 33 to 43)		1.40%	II
	NM_002944	33–43				
CDKN2A	NM_000077	1	c.35C > T	p.S12L	15.25%	II
CHEK2	NM_007194	11	c.1116_1117inv	p.K373E	8.36%	II
FLCN	NM_144997	11	c.1285dup	p.H429fs	2.82%	II
PMS2	NM_000535	11	c.1239dup	p.D414fs	2.96%	II
ACVR1	NM_001105	4	c.111_112dup	p.E38fs	2.08%	III
AKT3	NM_005465	8	c.739C > T	p.R247C	2.24%	III
APC	NM_000038	16	c.6386C > T	p.S2129L	2.63%	III
ASXL2	NM_018263	10	c.1037-1G > A	–	10.00%	III
EPHB4	NM_004444	7	c.1339C > T	p.P447S	9.10%	III
GNAQ	NM_002072	2	c.303C > A	p.Y101^a^	10.05%	III
HNF1A	NM_000545	4	c.865dup	p.G292fs	8.59%	III
KEL	NM_000420	9	c.1006G > A	p.V336M	2.04%	III
RTEL1	NM_032957	3	c.287_289del	p.A96del	2.96%	III
TCF7L2	NM_030756	14	c.1385dup	p.C463fs	5.25%	III
TET1	NM_030625	12	c.5531C > T	p.A1844V	2.15%	III
ZFHX3	NM_006885	10	c.10164_10166del	p.Q3389del	2.51%	III

^a^A premature stop codon due to a nonsense mutation

**Table 2:** Germline mutations in the patient

Gene	Transcript	Chromosome	Exon	Nucleotide change	Alteration	Homozygous/heterozygous	Clinical significance
ATRX	NM_000489	chrX	9	c.2806G > C	p.V936L	Homozygous	VUS
BCOR	NM_017745	chrX	4	c.935_937del	p.Q312del	Homozygous	Possibly benign
E2F3	NM_001243076	chr6	6	c.637G > A	p.G213R	Heterozygous	VUS
ETV1	NM_001163147	chr7	3	c.55G > A	p.G19R	Heterozygous	VUS
FLCN	NM_144997	chr17	14	c.1580G > A	p.R527Q	Heterozygous	VUS
FOXL2	NM_023067	chr3	1	c.118G > C	p.G40R	Heterozygous	VUS
KMT2A	NM_001197104	chr11	3	c.1512C > A	p.S504R	Heterozygous	VUS
LRP1B	NM_018557	chr2	34	c.5531G > A	p.G1844E	Heterozygous	VUS
PMS1	NM_000534	chr2	11	c.2440A > G	p.T814A	Heterozygous	VUS
PRDM1	NM_001198	chr6	2	c.170A > G	p.K57R	Heterozygous	VUS
RECQL4	NM_004260	chr8	5	c.520C > A	p.H174N	Heterozygous	VUS

*VUS* variant of uncertain significance

Besides in NSCLC, *ROS1* fusions have been identified in non-NSCLC solid tumors, such as brain tumors and gastrointestinal tumors. According to the study of Huang et al. (Huang et al. 2021), it seems *CD74* is a common partner of *ROS1* fusion in patients with NSCLC (49.8%), while a rare one in patients with non-NSCLC cases (4.9%). However, it has not been reported in patients with MPM. Crizotinib is a small-molecule inhibitor targeting ALK, MET, and ROS1 tyrosine kinases. To the best of our knowledge, the clinical efficacy of crizotinib in *ROS1*-positive MPM has not been reported.

In this case report, positive *ROS1* fusion was initially detected through RT-PCR in the patient. Subsequently, the partner *CD74* was determined using NGS. Of note, the patient responded very well to crizotinib for a durable time.

Crizotinib was the first oral targeted treatment approved for *ROS1*-positive advanced NSCLC. A long-term clinical benefit has been observed for the patients with *ROS1*-rearranged metastatic NSCLC since the application of crizotinib. However, the PFS of the patient in this case report (6.0 years) is far superior to the median PFS in Study OO12-01 (15.9 months), PROFILE 1001 (19.2 months), EUROS1 (9.1 months), EUCROSS (20.0 months), and METROS (22.8 months) (Landi et al. 2019; Mazieres et al. 2015; Michels et al. 2019; Shaw et al. 2019; Wu et al. 2018). Furthermore, the patient in this case report has survived for 7.6 years, which is longer than the median OS in Study OO12-01 (32.5 months) and PROFILE 1001 (51.4 months) (Shaw et al. 2019; Wu et al. 2018).

In this case report, the patient is an atypical case being only 19 years old. According to the patient’s dictation, his family has no history of malignant tumors, and he had no history of exposure to asbestos, therefore, this report might highlight the role of genomic testing particularly in those younger patients without an inherited/familial syndrome or no history of previous exposure to asbestos.

To the best of our knowledge, this case represents the first report of *CD74-ROS1* fusion identified in MPM. Meanwhile, it is also the first report of remarkable efficacy of crizotinib in a patient with MPM harboring *CD74-ROS1* fusion. Moreover, the patient obtained long-term clinical benefit from crizotinib. Therefore, this case illustrates the potential role of genomic testing and targeted therapy in selected cases of MPM.

## Data Availability

All of the data supporting the findings in this study are available upon reasonable request from the corresponding author (Weiming Huang).
